# Dissecting Breast Cancer Circulating Tumor Cells Competence via Modelling Metastasis in Zebrafish

**DOI:** 10.3390/ijms22179279

**Published:** 2021-08-27

**Authors:** Inés Martínez-Pena, Pablo Hurtado, Nuria Carmona-Ule, Carmen Abuín, Ana Belén Dávila-Ibáñez, Laura Sánchez, Miguel Abal, Anas Chaachou, Javier Hernández-Losa, Santiago Ramón y Cajal, Rafael López-López, Roberto Piñeiro

**Affiliations:** 1 Roche-Chus Joint Unit, Translational Medical Oncology Group, Oncomet, Health Research Institute of Santiago de Compostela, Travesía da Choupana s/n, 15706 Santiago de Compostela, Spain; ines.martinez.pena@rai.usc.es (I.M.-P.); pablo.hurtado.blanco@rai.usc.es (P.H.); Nuria.carmona.ule@sergas.es (N.C.-U.); carmen.abuin.redondo@sergas.es (C.A.); ana.belen.davila.ibanez@sergas.es (A.B.D.-I.); rafael.lopez.lopez@sergas.es (R.L.-L.); 2 CIBERONC, Centro de Investigación Biomédica en Red Cáncer, 28029 Madrid, Spain; miguel.abal.posada@sergas.es (M.A.); jahernan@vhebron.net (J.H.-L.); sramon@vhebron.net (S.R.y.C.); 3 Departamento de Zoología, Genética y Antropología Física, Facultad de Veterinaria, Universidade de Santiago de Compostela, 27002 Lugo, Spain; lauraelena.sanchez@usc.es; 4 Translational Medical Oncology Group, Oncomet, CIBERONC, Health Research Institute of Santiago (IDIS), University Hospital of Santiago de Compostela (SERGAS), Trav. Choupana s/n, 15706 Santiago de Compostela, Spain; 5 Translational Molecular Pathology, Department of Pathology, Vall d’Hebron Institute of Research (VHIR), Hospital Universitari Vall d’Hebron, Universitat Autònoma de Barcelona, 08035 Barcelona, Spain; achaachou@vhebron.net; 6 Department of Oncology, Complexo Hospitalario Universitario de Santiago de Compostela (SERGAS), 15706 Santiago de Compostela, Spain

**Keywords:** breast cancer, metastasis, circulating tumor cells (CTCs), CTC-clusters, zebrafish, in vitro models, in vivo models, cell survival

## Abstract

Background: Cancer metastasis is a deathly process, and a better understanding of the different steps is needed. The shedding of circulating tumor cells (CTCs) and CTC-cluster from the primary tumor, its survival in circulation, and homing are key events of the metastasis cascade. In vitro models of CTCs and in vivo models of metastasis represent an excellent opportunity to delve into the behavior of metastatic cells, to gain understanding on how secondary tumors appear. Methods: Using the zebrafish embryo, in combination with the mouse and in vitro assays, as an in vivo model of the spatiotemporal development of metastases, we study the metastatic competency of breast cancer CTCs and CTC-clusters and the molecular mechanisms. Results: CTC-clusters disseminated at a lower frequency than single CTCs in the zebrafish and showed a reduced capacity to invade. A temporal follow-up of the behavior of disseminated CTCs showed a higher survival and proliferation capacity of CTC-clusters, supported by their increased resistance to fluid shear stress. These data were corroborated in mouse studies. In addition, a differential gene signature was observed, with CTC-clusters upregulating cell cycle and stemness related genes. Conclusions: The zebrafish embryo is a valuable model system to understand the biology of breast cancer CTCs and CTC-clusters.

## 1. Introduction

Metastasis is a dynamic and complex multistep process involving the intravasation of tumor cells, spread to different organs and tissues, extravasation and cancer cell colonization, and final outgrowth of secondary lesions [1]. Metastases are the first cause of cancer-related deaths [2], and although significant advances have been made in terms of prevention, diagnosis, and treatment, the metastatic disease remains incurable. In breast cancer (BC), one out of ten newly diagnosed patients already have metastasis, and one-third will develop metastases within the first 5 years following diagnosis [3]. The progression from a localized to a metastatic disease has a poor prognosis, with a 5-year survival rate below 30% [4]. Thus, a major challenge in oncology research is to broaden the knowledge about the mechanisms of the metastatic process to develop clinical strategies to detect and treat metastatic patients.

During metastatic spread, tumor cells are released into the bloodstream, known as circulating tumor cells (CTCs), which are considered the precursors of secondary tumors. Thus, CTC enumeration has clinical relevance as the presence of CTCs in the blood of cancer patients is associated with a poor disease outcome in several cancer types, including BC [5,6]. CTCs can be found in the blood of cancer patients as single cells, or as small oligoclonal groups made up of a variable number of tumor cells (2->100 cells), called CTC-clusters. Although the frequency of CTC-clusters is low [7], preclinical data demonstrated that CTC-cluster have a higher metastatic potential than single CTCs, and their presence is associated with an earlier onset of metastasis [7,8,9,10]. Moreover, CTC-clusters seem to initiate the formation of polyclonal metastases [7,11,12,13], which suggests the existence of a mechanism of cooperation between cancer cell clones [14,15,16,17]. Few biological features of CTC-clusters are emerging showing the existence of strong cell–cell junctions within the tumor cells conferring a survival advantage in circulation and at distant sites [18,19], variable expression levels of mesenchymal and/or epithelial biomarkers [20], and the presence of phenotypic stem-cell traits [12,21]. The stem-like characteristics of CTC-clusters could partially explain their higher plasticity, as well as their increased ability to adapt to the microenvironment of the metastatic site when compared to single CTCs. The study of CTCs isolated from peripheral blood samples (known as a liquid biopsy) is a useful minimally invasive tool to identify the cellular and molecular mechanisms underlying the metastatic process [22]. However, the scarcity of CTCs and the rarity of CTC-clusters in the blood of cancer patients, added to the technical challenge that represents the detection, limits the advance in this area of research. In this work, we propose the use of “surrogate” models of BC CTCs to widen our understanding of the biology of CTC-clusters. The behavior of these CTC models will be studied by in vitro and in vivo xenograft assays. Traditionally, animal models of metastasis, and in particular mouse models, have been used to replicate the different steps of the metastatic cascade, allowing a better understanding of this complex process. Mouse tail vein injection experiments are the most commonly used to study the metastatic competence of CTCs [23]. In this sense, the zebrafish embryo is an interesting animal model to analyze the adaptation of tumor cells to different environments and their survival in circulation. Their fast extra-uterine development, transparency, and lack of an adaptive immune system make it an ideal in vivo tool to model metastasis [24]. Thus, few previous studies have shown the potential of the zebrafish to investigate the biology of CTC and CTC-clusters [25,26,27,28].

Here we describe the use of BC CTC-cluster models and the zebrafish embryo, in combination with in vitro and mouse experiments, to study the differential phenotypic traits of CTCs and CTC-clusters, explaining their metastasis-seeding ability. CTC-clusters derived from the epithelial MCF7 and mesenchymal-like MDA-MB-231 cell lines showed a differential behavior to single CTCs in in vitro assays of metastasis, reflecting their molecular subtype. Moreover, in zebrafish experiments, CTC-clusters showed a reduced invasion and dissemination capacity but enhanced survival and proliferation upon dissemination. The clustering of just a few cells was enough to increase the survival of tumor cells in circulation. Similar results were observed in tail vein injection experiments, where CTC-clusters resulted in a higher metastatic burden due to enhanced resistance to cell death. In addition, CTC-clusters displayed a higher resistance to shear stress measured in vitro, which translated into a higher metastatic competence in the zebrafish. Lastly, a gene expression analysis of tumor cells isolated from the zebrafish showed an upregulating of cell cycle and stemness-related genes in fish xenografted with CTC-clusters.

## 2. Results

### 2.1. Clusters of BC Tumor Cell Lines Show Differential In Vitro Behavior in Functional Assays Than Single Tumor Cells

**Figure 1:**
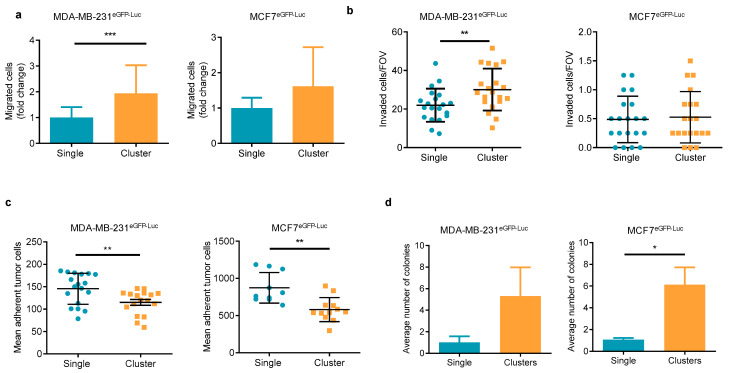
In vitro functional characterization of single CTC and CTC-cluster models. (**a**) Migration of single and cluster models derived from MDA-MB-231^eGFP-Luc^ (*n* = 6) and MCF7^eGFP-Luc^ (*n* = 4) cell lines. Data are expressed as the fold change of migrated cells relative to the migration observed in the single cell population; (**b**) Number of invaded MDA-MB-231^eGFP-Luc^ (*n* = 3) and MCF7^eGFP-Luc^ (*n* = 2) cells per field of view (FOV); (**c**) In vitro adhesion to endothelium of MDA-MB-231^eGFP-Luc^ (*n* = 4) and MCF7^eGFP-Luc^ (*n* = 2) cells. Data are expressed as the average of adhered cells per well; (**d**) Average number of colonies generated by MDA-MB-231^eGFP-Luc^ (*n* = 3) and MCF7^eGFP-Luc^ (*n* = 3) cells in the soft agar colony formation assays. Data are represented as the average number of colonies. * *p <* 0.05, ** *p <* 0.01, *** *p <* 0.001.

The human luminal BC MCF7 cells and the triple negative MDA-MB-231 cells were used to model the aggregation of tumor cells happening in CTC-clusters. Both cell lines stably expressed the enhanced green fluorescent protein (eGFP) and the luciferase gene (MCF7^eGFP-Luc^ and MDA-MB-231^eGFP-Luc^). Cells growing in monolayer at 80% confluence were dissociated with trypsin at different concentrations to obtain either an individual cell suspension or a clustered-cell suspension. Clusters of MCF7 cells maintained the characteristic expression of E-cadherin, while clusters of MDA-MB-231 cells maintained the expression of vimentin (Figure S1a). Equal numbers of cells from these cell suspensions were used in different in vitro functional assays representing different steps of the metastatic process, i.e., migration, invasion, adhesion to endothelium, and colony formation assays. In migration assays evaluating the initial stages of the metastatic process, clusters of the mesenchymal-like cells MDA-MB-231^eGFP-Luc^ showed an increased ability to migrate and invade in transwell in response to an FBS gradient compared to single cells (Figure 1a,b). Clusters of the epithelial cells MCF7^eGFP-Luc^ showed a trend for an increased migration than single cells, and no effect on cell invasion was observed, although as expected, the overall number of migrated and invaded cells was lower than the MDA-MB-231 cells (Figure 1a,b). We also evaluated the behavior of the cells in relation to their adherence to an endothelial cell monolayer mimicking the vasculature. Single and cluster populations were added over a monolayer of the EA.hy926 endothelial cells and incubated for a short period before washing off the non-adherent cells. Under these conditions, MDA-MB-231^eGFP-Luc^ and MCF7^eGFP-Luc^ single cells had a higher capacity to adhere to endothelial cells than clusters of cells (Figure 1c). In a soft agar colony formation assay mimicking the growth of tumor cells in an anchorage independent manner, clusters of MDA-MB-231^eGFP-Luc^ and MCF7 ^eGFP-Luc^ cells had an enhanced capacity to give rise to colonies than single cells (Figure 1d) with a larger size in the case of MCF7 ^eGFP-Luc^ (Figure S1b). Altogether, these results, besides showing the known differential behavior of these two BC cell lines, suggest that our model of clusters of tumor cells may be physiologically representative of CTC-clusters showing functional differences to individual cells.

### 2.2. Clusters of Tumor Cells Have a Lower Dissemination Capacity in the Zebrafish Embryo

We next wanted to investigate the metastatic potential of CTC-clusters using the zebrafish embryo xenograft model of BC metastasis. We initially tested the capacity of both MCF7^eGFP-Luc^ and MDA-MB-231^eGFP-Luc^ cells to metastasize in the zebrafish. Clusters of MDA-MB-231^eGFP-Luc^ cells injected into the perivitelline space of zebrafish embryos, near the convergence with the Duct of Cuvier and the pericardial space, had the ability to survive and disseminate to the fish tails (Figure S1c). On the other hand, the vast majority of xenografted clusters of MCF7^eGFP-Luc^ cells died the day after the injection, and only a low percentage of fish showed dissemination of MCF7^eGFP-Luc^ cells (Figure S1c), in agreement with previous reports [29]. Therefore, MDA-MB-231^eGFP-Luc^ cells were chosen for the following experiments.

**Figure 2:**
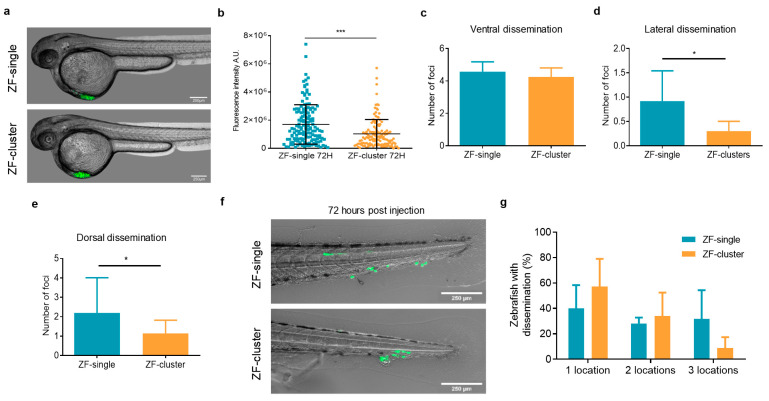
Functional characterization of single and cluster models in zebrafish xenografts. (**a**) Representative images of zebrafish showing tumor cell injection at the perivitelline space, near the convergence with the Duct of Cuvier (scale bar 250 µm); (**b**) Dot plot of the integrated fluorescence intensity in the tail of ZF-single and ZF-cluster 72 h after injection (*n* = 6 independent experiments). Each dot represents an individual fish; Number of foci found in the (**c**) ventral, (**d**) lateral, and (**e**) dorsal areas of the zebrafish tails; (**f**) Representative images of disseminated cells in the tails of ZF-single and ZF-cluster xenografts at 72 h post-injection (scale bar 250 µm); (**g**) Percentage of ZF-single and ZF-cluster xenografts with one, two, or three dissemination locations in the tail. * *p <* 0.05, *** *p <* 0.001.

CTC-clusters are found in the blood of BC patients in a much lower proportion than single CTCs. It is estimated that CTC-clusters comprise 1–30% of the total number of CTCs found in the blood of cancer patients and mouse models [30]. Based on these observations, we decided to investigate the dissemination capacity of single tumor cells and clusters in the zebrafish embryo. Equal numbers of single and clustered MDA-MB-231^eGFP-Luc^ cells (Figure S2a) were injected in the perivitelline space, near the convergence with the Duct of Cuvier and the pericardial space, of the zebrafish embryo (from now on referred to as ZF-single and ZF-cluster, respectively), and tumors of equal size were generated for both cell populations (Figure 2a and Figure S2b). The presence of disseminated cells in the fish tails was determined 72 h later based on fluorescence intensity quantification. Note that fish showing tumor cells in circulation after injection were excluded from our analyses. ZF-single showed a much higher number of CTCs in the tail 3 days post-injection than ZF-cluster (mean integrated intensity of 1.34 × 10^6^ for single cells vs. 7.09 × 10^5^ for clusters) (Figure 2a). This result may suggest that single cells had a significantly higher capacity to abandon the injection site and disseminate through the circulatory system of the zebrafish embryo. We also wanted to evaluate the capacity of single CTCs and CTC-clusters to migrate once disseminated to the fish tail. To this end, we determined the presence of tumor cell foci in different locations of the fish tail, named as ventral, lateral, and dorsal, corresponding to the distribution of the vasculature (see Figure S2c and methods). Representative images of the dissemination of cells in ZF-single and ZF-cluster are shown in Figure S2d. The average number of foci per fish found in the ventral area did not differ between ZF-single and ZF-cluster (4.5% vs. 4.2%, respectively; Figure 2b), as a very large proportion of fish on both populations showed cells arrested in this region (88.2% of ZF-single and 95.6% of ZF-cluster; Figure S2d). CTCs in the circulation of the fish are dragged by the direction of blood flow from the site of injection along the dorsal aorta towards the cardinal vein, where they arrest at the caudal plexus [27]. However, significant differences were found in the average number of foci found at the lateral (Single 0.9 ± 0.6 vs. Cluster 0.3 ± 0.2; *p <* 0.05) and dorsal (Single 2.1 ± 1.8 vs. Cluster 1.1 ± 0.6; *p <* 0.05) areas of the tails, with the single cell population generating more foci than clustered cells at both locations (Figure 2c–e). These results were also accompanied by a higher percent of ZF-single that showed disseminated CTCs at the lateral and dorsal regions compared to ZF-cluster (37.0% ± 18 vs. 15.07% ± 13.4 and 63.2% ± 16.9 vs. 37.8% ± 13.2, respectively; *p >* 0.05) (Figure S2f,g), as well as a higher percentage of fish in which CTCs reached the three locations (Figure 2f). Altogether, these data indicate that CTC-clusters have lower dissemination capacity in the fish as well as an impaired capacity of movement once disseminated, probably due to physical restrictions related to their size.

### 2.3. Disseminated Clusters of Tumor Cells Survive Better in the Zebrafish Than Single Cells

**Figure 3:**
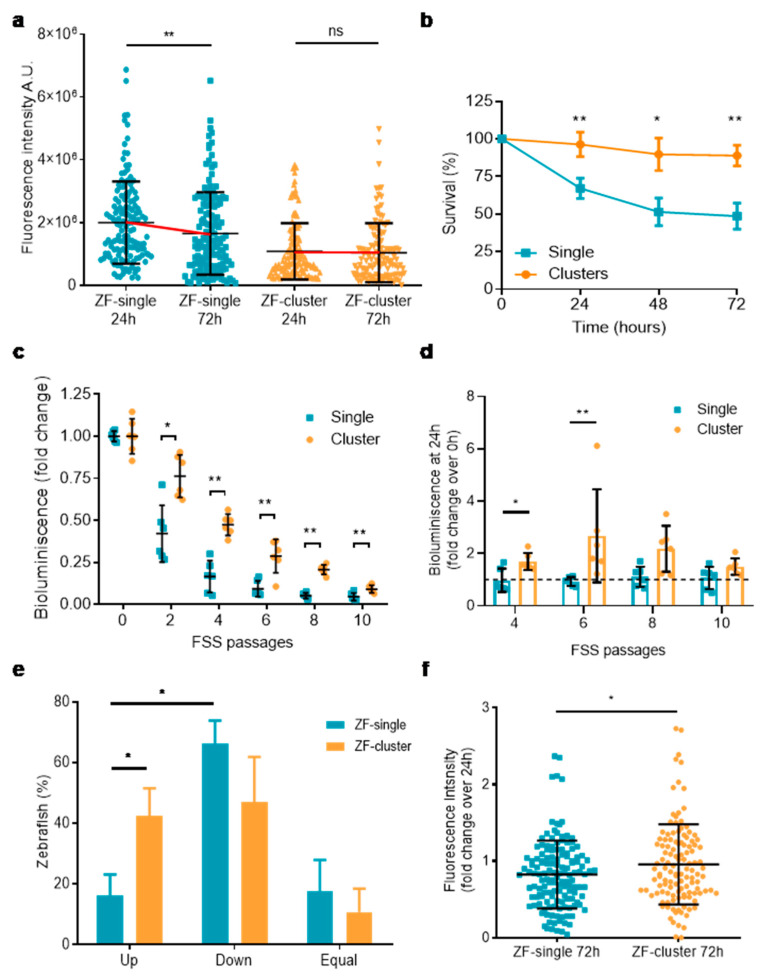
Time course of cell dissemination to the tail in the ZF-single and ZF-cluster xenografts. (**a**) Dot plot of the integrated fluorescence intensity of ZF-single and ZF-cluster, at 24 and 72 h post-injection (*n* = 6 independent experiments). Each dot represents an individual fish; (**b**) Cell survival in the ZF-single and ZF-cluster xenografts over time. Data are expressed relative to the initial survival observed at 0 h post-injection (*n* = 4); (**c**) Bioluminescent signal throughout fluid shear stress (FSS) assay. Data are represented relative to the non-FSS exposed control (P0), (*n* = 3); (**d**) Bioluminescence fold change 24 h after the exposure to FSS. Data are expressed relative to the bioluminescent signal registered after finishing the FSS cycles (0 h; dotted line) (*n* = 3); (**e**) Percentage of ZF-single and ZF-cluster xenografts, whose fluorescence in the tail has increased, decreased, or maintained over time (*n* = 6 independent experiments); (**f**) Integrated fluorescence intensity fold change of ZF-single and ZF-cluster xenografts. Data are normalized by the integrated fluorescence intensity observed at 24 h post-injection. * *p <* 0.05, ** *p <* 0.01, ns not significant.

The higher dissemination of tumor cells in ZF-single compared to ZF-clusters could be explained by an enhanced capacity of single CTCs to form metastases or by a higher ability to abandon the site of injection (the primary tumor). To investigate these possibilities, we performed a time-course experiment in which we analyzed the presence of cells in the tails at 24 and 72 h post-injection. This analysis revealed that already at 24 h the number of CTCs disseminated in the tails of ZF-single was significantly higher than the number of CTCs in ZF-clusters (mean integrated fluorescence 1.99 × 10^6^ ± 1.3 × 10^6^ vs. 1.08 × 10^6^ ± 8.98 × 10^5^, respectively; *p <* 0.0001) (Figure 3a), suggesting that single cells have a higher ability to abandon the site of injection over clusters of cells. Importantly, we observed that while in ZF-single the fluorescence signal significantly decreased from 24 h to 72 h (from 1.99 × 10^6^ ± 1.3 × 10^6^ to 1.65 × 10^6^ ± 1.31 × 10^6^; *p <* 0.01), in ZF-cluster it remained the same (1.08 × 10^6^ ± 9.4 × 10^5^ vs. 1.04 × 10^6^ ± 9.4 × 10^5^; *p >* 0.05) (Figure 3a). These results seem to indicate that, although clusters of tumor cells are shed in a lower proportion from the xenograft site than single cells, those clusters may survive better in the circulation or when arrested in the zebrafish tail, ruling out the possibility of the enhanced metastasis-seeding ability of single CTCs.

However, our experimental setup did not allow us to tell whether the increased survival of CTCs observed in ZF-cluster over ZF-single is an intrinsic feature of the CTC-clusters or due to the aggregation of different clusters in the zebrafish circulatory system (random aggregation among cells arrested in the ventral area of the fish). To further investigate this, small numbers of cells as single cells or clusters were injected near the pericardial space of zebrafish embryos and followed on time to determine the number of surviving tumor cells in each fish. We observed a decrease in the number of surviving cells injected as single cells over time, while survival of the cells was maintained when injected as clusters (Figure 3b). The individual experiments with the specific numbers of cells injected into the fish are shown in Figure S3a and representative images of the cells inside the fish in Figure S3b. This result indicates that small-size clusters of tumor cells (2 to 4 cells) in the circulation survive better than individual cells, suggesting that the clustering of as low as two or three cells is enough to enhance tumor cell survival against circulatory forces. Therefore, our data points towards an intrinsic survival advantage of CTC-cluster in circulation derived from the physical interaction of tumor cells. Moreover, these results were supported by an in vitro fluidic assay designed to evaluate the resistance of tumor cells to fluid shear forces (FSS) [31,32]. Suspensions of single and clustered MDA-MB-231^eGFP-Luc^ cells loaded in a syringe were repeatedly subjected to high magnitude (>1000 dyn/cm^2^) FSS passages (P0–P10) using a syringe pump, and their viability was assessed by the bioluminescence signal every two passages. As expected, FSS passages caused cell death in both populations; however, this effect was significantly less pronounced in the suspensions of clusters (Figure 3c). After P2 the viability of single and clustered cells was reduced in a 58% and 24% respectively, and an 84% and 53% after P4, with respect to the viability of the cells at P0 (not exposed to FSS) (Figure S3c). These results altogether indicate that the enhanced survival of CTC-clusters in the zebrafish is at least in part due to intrinsic resistance of CTC-clusters to shear stress, the main cause of tumor cell death in the circulation [33].

### 2.4. Clusters of Tumor Cells have a Proliferative Advantage over Single CTCs

Previously, a link between the clustering of CTCs and a higher proliferation at the metastatic site has been established; therefore, we wanted to investigate this possibility. We looked at the proliferative state of the tumor cells upon FSS. Cells from single cell and cluster suspensions subjected to P4–P10 FSS passages were collected and plated in tissue culture wells for 24 h, the time after which bioluminescence was measured and compared to time 0 h. The bioluminescence signal from single cell suspensions collected after FSS P4–P10 passages 24 h later did not differ from the signal measured at 0h, indicating the lack of cell proliferation in the culture. However, the bioluminescence signal from clustered cells suspensions increased at P4, P6, and P8 relative to time 0h, indicating the presence of a higher number of cells in the culture, and therefore cell proliferation (Figure 3d). Pictures of the cultures reflect a higher number of cells, a lower amount of cell debris, as well as a larger number of cells adhered to the surface of the well in the cluster population compared to single cell population (Figure S3d).

This result led us to evaluate the proliferation of CTCs and CTC-clusters in the zebrafish model. We compared the integrated fluorescence intensity values obtained at 24 and 72 h for each fish included in the previous time-course analysis, allowing us to determine the proportion of ZF-single and ZF-cluster in which the number of disseminated CTCs in the tails is either increasing or decreasing over time. The percent of fish in which the fluorescence intensity increased from 24 to 72 h (meaning cell proliferation) was significantly higher in the ZF-cluster population than in ZF-single (39.4 ± 7.3% vs. 16.2 ± 6.89%, respectively; *p* < 0.05) (Figure 3e). In agreement with this result, we observed a tendency towards a reduction in the percent of ZF-cluster in which the fluorescence decreased compared to ZF-single (52.6 ± 9% vs. 66.1 ± 7.6%, respectively; *p* > 0.05), suggestive of cell death. A similar tendency was observed in the percent of fish in which the fluorescence did not change between time points (CTC-clusters 7.9 ± 5.5% vs. Single CTCs 17.5 ± 10.3%; *p* > 0.05) (Figure 3e). Moreover, there was a significantly higher proportion of ZF-single in which the fluorescence decreased rather than increased, which was not observed in ZF-cluster (Figure 3e). In addition, the fold change on the fluorescence intensity from 24 to 72 h was significantly increased in the ZF-cluster population compared to the single cell population (Figure 3f). Taken together, these data indicate that disseminated CTCs in clusters have not only an enhanced survival but also a proliferative advantage over individual CTCs, allowing them to potentially grow into larger lesions due to an interplay between reduced cell death and a higher proliferation.

### 2.5. CTC-Clusters Have an Enhanced Survival and Metastatic Potential in Mice Compared to Single CTCs

**Figure 4:**
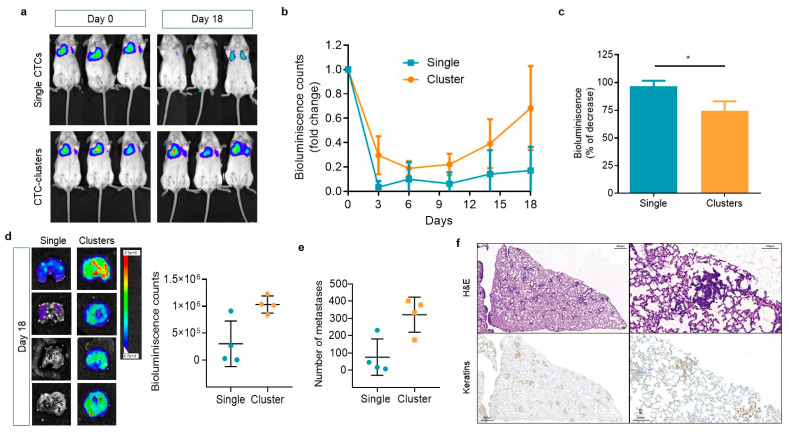
Assessing metastatic colonization ability of single and cluster models in the mouse. (**a**) Representative images of the development of lung metastatic lesions over time in mice injected in the lateral tail vein with single CTCs or CTC-clusters assessed by measuring the bioluminescence signal (counts); (**b**) Bioluminescence signal throughout time emitted by the tumor cells that has colonized the pulmonary tissue (*n* = 4 per group); (**c**) Percentage of decrease of the bioluminescence signal in the lung of mice 3 days after tail vein injection of single and cluster populations (* *p* < 0.05); (**d**) Representative pictures of the bioluminescence signal measured in the surgically removed lungs of mice injected with single CTC or CTC-cluster models, and quantification of the bioluminescence signal; (**e**) Quantification of the number of metastatic foci in the lungs of mice injected with single CTCs or CTC-clusters; (**f**) Representative images of lungs with metastases, visualized with Hematoxin-eosin (H&E, upper panels), and sections of the lung stained with an antibody for keratins (bottom panels). Note the brown staining in tumor clusters and nodules. Left images are shown at a low magnification (×5; scale bar 500 µm), and right images at a high magnification (×20; scale bar 1000 µm).

To determine whether CTC-clusters have similar behavior in a mammalian model, we injected equivalent numbers (5 × 10^5^ cells/mice) of MDA-MB-231^eGFP-Luc^ cells, prepared either as clusters or as dissociated individual cells, into the lateral tail vein of immunodeficient mice. Lung colonization and metastasis formation by tumor cells were evaluated along time by bioluminescence signal imaging (Figure 4a). As expected, we observed that the bioluminescence signals in the lungs of mice from clustered and single tumor cells drastically decreased during the first week compared to the moment of injection (Figure 4b). On day 3 post-injection, the bioluminescence signal dropped 96% for single CTCs compared to 76% for CTC-clusters (Figure 4c). This result is in agreement with the zebrafish and FSS experiments and indicates that clusters of tumor cells have a survival advantage over single cells upon dissemination through the circulation to secondary organs. Moreover, follow-up of tumor development in the lungs at day 14 post injection showed that clustered tumor cells began to proliferate again, and metastatic growth was observed (Figure 4b). This effect was even more pronounced from day 14 to day 18, the time at which the experiment was stopped. On the contrary, single cells showed a delayed and limited metastatic growth compared to tumor cell clusters (Figure 4b). This result was confirmed by the bioluminescence signal measured in the extracted lungs of these mice, which was higher in those injected with clusters (Figure 4d). Moreover, the histological analysis of the lungs confirmed the higher incidence of metastases in mice injected with CTC-clusters (Single CTCs 75.5 ± 105 vs. CTC-cluster 321.3 ± 101; *p* = 0.057) (Figure 4e,f). Altogether, these results indicate that CTC-clusters have enhanced survival and proliferative capacity and metastatic potential compared to single CTCs. Importantly, data gathered in the zebrafish could be reproduced in the mice, indicating that the use of zebrafish to model metastasis can be a valuable tool to understanding the biology of CTCs, and particularly of CTC-clusters.

### 2.6. Gene Expression Profiling Supports the Metastatic Features of CTC-Clusters

**Figure 5:**
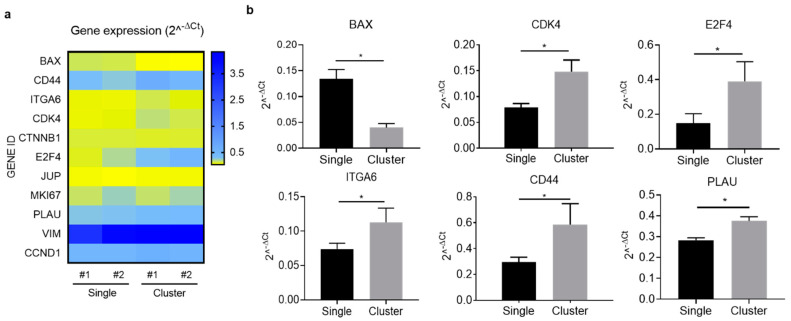
Gene expression profile of the single and cluster cells disseminated in the tail of ZF xenografts. (**a**) Heatmap of the expression levels of disseminated cells in the ZF-single (Sing#1, and Sing#2) and ZF-cluster (Clust#1, and Clust#2) xenografts expressed as 2^−ΔCt^; (**b**) Relative mRNA expression observed in the disseminate cells of the ZF-single and ZF-cluster xenografts. Data are expressed relative to the average expression levels of β-2-microglobulin (β2M), and Glyceraldehyde-3-Phosphate Dehydrogenase (GAPDH), which were used as the housekeeping genes (* *p* < 0.05).

Lastly, we wanted to investigate the molecular mechanisms supporting the biological features of CTC-clusters in the zebrafish model. Given the low number of tumor cells disseminated to the fish tails, we decided to carry out a comparative gene expression assay in pools of cell suspensions derived from ZF-single and ZF-cluster of four independent experiments obtained after the digestion of the fish tails 48 h after microinjection. This strategy would allow us to increase the amount of tumor-derived material in the samples. We analyzed a panel of genes involved in proliferation/cell cycle, survival, and stemness (Table S1). Interestingly, we observed a differential gene expression pattern between ZF-single and ZF-cluster cells in some genes (Figure 5a and Figure S4a). The pro-apoptotic gene BAX was significantly downregulated in ZF-cluster (*p* < 0.05), and the cell cycle regulators CDK4 and E2F4 were upregulated (*p* < 0.05), accompanied by a not significant increase in the expression of CCND1 (*p* = 0.057) (Figure S5b), the gene encoding the cyclin D1. However, no differences were observed in the expression of MKI67, encoding the proliferation marker Ki-67 (Figure S4b). In addition, ITGA6 and CD44 genes, related to the stem cell-like features of BC cells, were upregulated in the cluster population (*p* < 0.05) (Figure 5b). Moreover, PLAU, the gene encoding the plasminogen activator, also showed a higher expression in cells from ZF-clusters (Figure 5b). No differences between populations were found in the expression of CTNNB1, JUP, and VIM (Figure S4b). These results point towards a gene expression profile supporting a pro-survival and proliferative phenotype in CTC-clusters.

## 3. Discussions

The scarcity of CTC-clusters in the blood of cancer patients represents an important hurdle in the study of their biology, limiting our biological knowledge about their enhanced metastatic potential. Taking advantage of cellular models mimicking BC CTC-clusters and of the zebrafish and mouse as biological systems, we show that clusters of CTCs have an intrinsic enhanced survival and proliferation advantage over single CTCs, allowing them to better survive while in circulation and to proliferate giving rise to metastatic lesions. Furthermore, we show that despite the reduced capability of CTC-clusters to abandon the primary tumor (xenograft site) and migrate through the zebrafish body, once disseminated, their enhanced resistance to shear stress allows them to recover a proliferative state. These biological features were supported by a gene expression profile showing the upregulation of pro-survival, cell cycle, and stemness-related genes in the fish xenografts. Lastly, we also show the zebrafish embryo as a valuable model system to understand the biology of CTCs and CTC-clusters, allowing us to obtain findings similar and compatible to those obtained in the mouse.

Several factors are hampering the research on the biology of CTCs and CTC-clusters and their role in the complex process of metastasis: The low number of CTCs and the significantly lower frequency of CTC-clusters in a blood drawn from a cancer patient, the challenge of the ex vivo expansion of CTCs in cultures or in mice, and the technological limitations for the capture, isolation, and imaging of CTCs and CTC-clusters from and in the circulation. To overcome some of these limitations, we decided to use the phenotypically different MCF7 and MDA-MB-231 human BC cell lines to generate in vitro “surrogates” of tumor cell clusters and the zebrafish embryo as a biological system to model metastasis. Our protocol to generate clusters allows the maintenance of cell-to-cell unions and the expression of the epithelial and mesenchymal markers E-cadherin and vimentin (Figure S1a). Our experimental results from in vitro and in vivo assays not only reflect the phenotypic differences between these two cell lines, but also show a clear difference between their behavior when presented as single or as small cell clusters. Similarly, other research groups have previously proven the value of using BC and other tumor types cell lines to study the biology of CTC-clusters [7,12,34]. On the other hand, we chose the zebrafish as this model provides biologically relevant information about the metastatic process, but also because it allows gaining insight into the behavior of the tumor cells in circulation [26,27,28,35,36].

An important feature of the zebrafish model regarding the shedding of CTCs and CTC-clusters to the circulation is that it was able to reproduce the low frequency of CTC-clusters found in the blood of cancer patients and mouse models compared to single CTCs [30]. The data shows that the day after cell injection the volume of CTCs found away from the injection site in fish injected with single cells was two-fold higher than in the fish injected with clusters of tumor cells. This difference is probably an underestimation since the analysis on the disseminated cells cannot discriminate between a cluster that has migrated away from the tumor (xenograft) and a cluster formed by migrated single cells that have aggregated within the fish vasculature. Moreover, it should be noted that some degree of single cells are always present in the cell cluster suspensions due to the nature of the protocol for the generation of clusters. In any case, xenografts of tumor cell clusters generated in the fish shed a lower number of cells into the circulation, suggesting that there might be an active mechanism of migration/invasion towards the circulatory system of the zebrafish, probably involving a collective cell migration phenomenon as observed in other animal models [11].

Our experimental in vitro assays indicate that clusters of tumor cells have an increased ability to migrate, invade, and form colonies compared to individual tumor cells. Although the result on cell migration might seem counterintuitive, in response to a gradient, small clusters can move towards each other and aggregate into larger clusters, increasing migration velocity [37]. On the other hand, the enhanced colony formation ability of tumor cell aggregates has been previously reported, and it is believed to be due to the lower levels of apoptosis and mediated by a molecular signaling promoting anchorage-independent survival [38].

An important step in the metastatic process is the adhesion of tumor cells to the vascular endothelium previous to the extravasation. In vitro endothelium adhesion assay indicates that clusters of BC cells do not adhere as efficiently as single cells to the endothelial cell monolayer. However, the static nature of the assay may prevent the low affinity and transient interactions with the endothelium that are likely to facilitate the phenomenon of cell rolling of tumor cells, similar to immune cells [39,40], probably to detriment of the cluster population which exposes a larger surface of cell membrane than single cells. This in vitro scenario is very different from that experienced by CTCs and CTC-clusters in the zebrafish circulation, where the hemodynamic forces of the blood flow contribute to the arrest and the adhesion of tumor cells to the endothelium and drive tumor metastasis [27]. Indeed, the arrest of CTC-clusters seems to be favored by physical occlusion [7,11,41]. In line with these observations, our result clearly shows that the largest proportion of cell foci from both cell populations were found at the ventral region of the fish, mainly located in the caudal vein plexus, the preferential location for the arrest of CTCs [25,27]. CTC-clusters were also occasionally found at the dorsal (dorsal longitudinal anastomotic vessel) and lateral (intersegmental vessels) locations, although at a lower percentage of fish and much lower number than single CTCs. The ability of clusters to reach those areas is probably due to the demonstrated capability to traverse small-size capillaries, by reorganizing themselves into single-file chains which allow reducing their hydrodynamic resistance [25]. Based on this, we could interpret that the dissemination of CTC-clusters is physically restricted by their size, allowing their quick entrapment in small vessels, and as a consequence, reducing the time in circulation [7]. This per se could be an advantage of CTC-clusters over CTCs in the process of metastasis. Some data is starting to emerge in this regard, showing that the high cellularity of the clusters might favor successful extravasation and enhance metastatic potential [27,28].

The presence of CTC-clusters in the blood of cancer patients is associated with a poorer clinical outcome [5,7,8,10,42,43,44,45,46,47], and emerging key biological features could explain their increased metastasis-initiating capacity. Thus, the presence of functional cell–cell junctions, cytoskeletal proteins, and molecular interactions can promote a survival advantage to modify the expression of genes associated with stemness and proliferation [11,12,21]. Our data on the zebrafish model corroborate it and point to an intrinsic survival advantage of the cluster, derived from the clustering of tumor cells. The increased survival seems to be conditioned by the interaction of tumor cells occurring in the initial cell cultures, but in cancer patients, this might be the result of the aggregation of tumor cells in the primary tumor as a cluster [7,11] or the aggregation of individual CTCs in the circulatory system [12]. Indeed, our zebrafish data shows that the clustering of just a very few cells (only two to four cells) is enough to enhance survival and protect them from cell death against fluid shear stress and during the transit in circulation. Cell–cell interactions within the cluster protect from anoikis, a form of cell death derived from the loss of adhesion-dependent survival signals by epithelial cells when transitioning in the bloodstream [48]. Moreover, clinical evidence indicates that the incidence of apoptotic CTCs in clusters isolated from the blood of patients with small-cell lung cancer and breast cancer is almost anecdotal [10,42]. Our results are in agreement with these data and show a significantly reduced incidence of cell death in clustered cells compared to the single cells. These results add value to the zebrafish as a model to study the biology of CTC-clusters, as it allows for the detailed study of the viability of CTCs and CTC-clusters arrested in the blood capillaries, something quite difficult to assess in cancer patients.

Studies with mouse models of breast and other cancer types have shown that CTC-clusters have an increased metastasis-initiating potential compared to single CTCs [7,11,12,13,49]. Clusters of breast cancer cells injected in mice are more resistant at distal metastatic sites to apoptosis than individual CTCs, allowing them to expand more rapidly and forming larger tumors than individual cells [7]. Our mice experiments reproduced these data and showed a significantly lower incidence of cell death and a faster growth of metastatic lesions in the lungs of mice injected with CTC-clusters than with single CTCs, indicating a higher degree of proliferating tumor cells. In line with this, the in vitro FSS assay showed that the number of cells in the cultures of CTC-clusters subjected to various passages of FSS increased in just 24 h, while this effect was not observed in the cultures derived from single cell suspensions. In addition, the time-course analysis of zebrafish experiments showed a significantly higher percentage of fish in which the fluorescence increased over time in the ZF-cluster population compared to the ZF-single population. This result may indicate that the number of disseminated cells in a proliferative state is higher in the CTC-clusters. Moreover, tumor cells extravasated in zebrafish as multicellular clusters through angiopellosis exhibit an augmented ability to proliferate compared to individually extravasating cells, which remain dormant at a higher frequency [28]. These clusters also exhibit an increased ability to form tumors at distant sites in mice, suggesting that CTCs maintain the ability to exit vessels as clusters in mammalian vasculature [28]. Although our zebrafish and mouse experiments did not assess the degree of extravasation between CTC-clusters and single CTCs, this evidence goes in line with the proliferative state of CTC-clusters observed in vitro (FSS assay) and in zebrafish and mice experiments. Therefore, it would be easy to speculate that the increased proliferation of CTC-clusters in our experiments derives from extravasation by angiopellosis, contributing to their higher efficiency at metastases formation. Altogether, these results point towards a higher resistance of the CTCs in the clusters to cell death and easy recovery of the proliferative state upon exiting the vasculature, all of which allows for an increased metastasis-initiating potential.

Interestingly, the gene expression analysis of tumor cells isolated from zebrafish (ZF-single and ZF-cluster) shows a differential profile between both tumor cell populations, and points towards an upregulation in CTC-clusters of genes involved in cell survival (PLAU), cell cycle regulation (CDK4 and E2F4), and in stemness (ITGA6 and CD44). PLAU, the gene codifying for the Urokinase-Type Plasminogen Activator (UPA), plays an important role in the proteolytic degradation of the ECM, and it is known to protect against cell death [50,51,52]. Therefore, the upregulation of PLAU in CTC-clusters may be a mechanism to enhance CTC survival in circulation and might suggest a role in tumor cell clustering. However, preclinical data suggest that urokinase exerts antimetastatic effects by dissociating CTC-clusters [53]. In the light of these findings, our results grant further studies investigating the role of urokinase in CTC clustering and metastatic seeding ability, as urokinase treatment may also increase the risk of tumor cell invasiveness and metastatic spreading [54]. On the other hand, we observed an increased expression of CDK4 and E2F4 in tumor cells isolated from ZF-cluster, genes regulating cell cycle progression. The CDK-cyclin D pathway participates in cell cycle control and mediates G1-S phase transition and it is commonly deregulated in cancer [55]. CDK4 expression is increased in TNBCs and correlates with poor overall survival and relapse-free survival [56]. Particularly, the triple negative MDA-MB-231 cell line is sensitive to CDK4/6 inhibition, leading to suppressed proliferation and promoted apoptosis [56]. Thus, in our model of metastasis, CDK4 might drive cell cycle progression and the proliferation of CTC-clusters. Regarding E2F4, our data suggest that high E2F4 expression acts as a positive regulator of CTC-cluster proliferation. However, in BC, this transcription factor has been proposed to act as an oncogene [57] and a tumor suppressor [58,59]. Besides, its activity, rather than expression, has been shown to be a prognostic factor in BC [60]. Our data, only based on gene expression, does not allow us to evaluate the activity of the transcription factor and its implication in CTC-cluster proliferation. In addition, we observed the upregulation of CD44, a stem cell marker involved in BC cell clustering, survival, invasiveness, and metastasis formation [12]. Interestingly, CDK4 may act as a stem cell regulator by modulating the expression of CD44 and CD24 in BC cells as the CDK4 inhibitor Palbociclib decreased the fraction of CD44+/CD24− stem cells in MDA-MB-231 cells [61]. Therefore, our results suggest a controlled mechanism by which CTC-clusters upregulate CDK4, cyclin D1 and CD44 to increase proliferative and stemness features, allowing them to survive and grow into metastatic lesions. In line with the stem-like phenotype found in CTC-clusters, we also observed the increased expression of ITGA6 (also known as CD49f), a cell-surface protein involved in cell adhesion described as a marker to enrich for BC stem cells [62]. High expression of ITGA6 has been associated with an enhanced invasion and tumor-initiating capacity in an MDA-MB-231 model of metastatic BC [63]. A study in mouse xenografts derived from ER-negative breast tumors identified a population of cells with xenograft initiating capacity defined by the presence of CD44-positive and CD49f high cells, together with the high expression of CD133/2 [64]. Importantly, CTC-clusters isolated from the blood of BC patients possess stem-cell-like molecular features compared with single CTCs [12,21]. Therefore, altogether our results in the zebrafish model indicate that the gene expression profile observed in tumor cells isolated from ZF-clusters, as opposed to ZF-single isolated cells, supports a proliferative and stem-like phenotype favoring a metastasis initiation capacity. Moreover, they present the zebrafish metastasis model as a valuable tool to decipher the biology of CTC-clusters.

## 4. Materials and Methods

### 4.1. Cell Culture and Generation of Single and CTC-Cluster In Vitro Models

In vitro models of both single CTCs and CTC-clusters were generated using the human triple-negative BC cell line MDA-MB-231 and the luminal A BC cell line MCF7. Both cell lines stably expressed the enhanced green fluorescent protein (eGFP) and the luciferase gene (Luc) (MDA-MB-231^eGFP-Luc^ and MCF7^eGFP-Luc^). MDA-MB-231^eGFP-Luc^ cells were purchased from Tebu-Bio (Barcelona, Spain) and MCF7^eGFP-Luc^ were purchased from GeneCopoeia, Inc (Rockville, MD, USA). Cells were cultured in DMEM High Glucose supplemented with 10% fetal bovine serum (FBS) and 1% *v/v* Penicillin/Streptomycin (P/S), at 37 °C in a humidified atmosphere containing 5% CO_2_. According to the experimental needs, two different protocols were used for the generation of single and CTC-cluster models: (i) The required number of cells was cultured overnight in low-adherence conditions in order to allow cell aggregation. The day after, cell suspensions were physically dissociated by gentle pipetting to obtain a single-cell suspension and a small cell-group suspension, corresponding to single CTC and CTC-cluster models, respectively; (ii) Adherent cell monolayers at an 80% confluence were dissociated with different concentrations trypsin-EDTA, 10× (0.25% *v/v*) and 1× (0.05% *v/v*), to obtain either an individual cell suspension or a clustered-cell suspension, respectively.

### 4.2. Transwell Migration and Invasion Assays

Migration of single cells and clusters was evaluated by using 6.5 mm Transwell^®®^ with 8.0 μm Pore Polycarbonate Membrane Insert (Corning, Madrid, Spain). Cells were cultured overnight under suspension and serum starvation conditions. For invasion assays, each Transwell was coated with Growth Factor Reduced Matrigel (Corning) and incubated for 3 h to allow Matrigel polymerization. Cells were cultured overnight under suspension and serum starvation conditions. The day after, 5 × 10^4^ cells/transwell were seeded as both single and cluster suspension. Assays were incubated with a 10% gradient of FBS (37 °C, 5% CO_2_), during 8 h for MDA-MB-231 cell line, or 24 h in the case of MCF7 cells. After the incubation, transwell membranes were scraped in order to remove the non-migrated cells. Migrated cells were fixed with 3% w/v PFA + 1% *v/v* glutaraldehyde (Sigma-Aldrich, St. Louis, MO, USA) for 15 min. Migrated eGFP-expressing cells were counted using a microscope Leica DMi8 (Leica Microsystems, L’Hospitalet de Llobregat, Spain) and the free software ImageJ (NIH Image, Bethesda, MD, USA).

### 4.3. Adhesion to Endothelium Assay

Endothelial cells EA.hy926 were purchased from ATCC (USA) and cultured in DMEM High Glucose supplemented with 10% FBS and 1% P/S. EA.hy926 (5 × 10^4^ cells/well) cells were seeded in 96-well multiwell plates previously coated with gelatin. EA.hy926 cells were cultured at 37 °C, 5% CO_2_ for 24–48 h in order to obtain a cell monolayer. Then, tumor cells (2.5 × 10^4^ cells/well; six replicates per condition) were added as single or cluster cell suspension over the endothelial cell monolayer in serum starvation conditions and incubated for 45 min to allow the adhesion to the endothelium. After incubation, non-adhered tumor cells were removed and the wells were washed with Phosphate Buffered Saline (PBS) and fixed with 4% *w/v* paraformaldehyde (PFA). The number of tumor cells adhered to endothelium was determined by using the microscope Leica DMi8.

### 4.4. Soft-Agar Colonogenesis Assay

Cells (1 × 10^4^ cells/well) either as single cells or clusters were suspended in 0.3% *w/v* molecular grade agarose (Fisher BioReagents, Madrid, Spain), penicillin/streptomycin, 10% FBS in DMEM and layered on top of a previously solidified 1.5 mL 0.5% *w/v* agarose, P/S, 10% FBS in DMEM in 6-well multiwell plate. Cells were allowed to grow at 37 °C, 5% CO_2_ for a period of 3-4 weeks. Colonies growing in the agarose were fixed and stained using 0.005% *w/v* crystal violet (Sigma-Aldrich, St. Louis, MO, USA) in methanol, for 1 h at RT, and with shaking. The number of colonies were counted and the area of each colony was determined using a Leica DMi8 microscopy (Leica Microsystems, L’Hospitalet de Llobregat, Spain).

### 4.5. Immunofluorescence

Approximately 1 × 10^4^ cells in suspension were washed with PBS, centrifuged in a Cytospin 4 Centrifuge (Thermo Scientific, Waltham, MA, USA) at 350 rpm for 10 min, and fixed over microscopy slide with 4% *w/v* PFA for 15 min. After fixation, cells were washed with PBS and incubated with the InsidePerm Buffer (Inside Stain Kit, Miltenyi Biotec, Madrid, Spain) for permeabilization and as antibody diluent. Immunostaining was done incubating with the antibodies against E-Cadherin (1:200, ab40772; Abcam, Cambridge, UK) and Vimentin (D21H3) XP^®®^ Rabbit mAb (1:100, #5741; Cell Signalling, Danvers, MA, USA) for 1 h at RT. After incubation with the primary antibodies, cells were washed and incubated with the secondary antibody Anti-Rabbit AlexaFluor 647 (1:1000, ab150079; Abcam, Cambridge, UK). Nuclei were stained with DAPI. Fluorescence images were acquired with a DMi8 microscopy (Leica).

### 4.6. Zebrafish Xenograft and Micromanipulation Experiments

Wild type zebrafish (*Danio rerio*) embryos were generated by natural mating of adult fish. Embryos were incubated with PTU (N-Phenylthiourea, Sigma) at a 0.003% *w/v* concentration in order to remove surface pigmentation, and maintained at 28 °C. For xenograft experiments, two days postfertilization zebrafish larvae were de-chorionized and anaesthetized with 0.003% tricaine (Sigma) *w/v* in E3 medium with PTU, and positioned on a 10 cm Petri dish coated with 1.5% *w/v* agarose. Immediately prior to injection, single and clustered cell suspensions of MDA-MB-231^eGFP-Luc^ and MCF7^eGFP-Luc^ were generated from the adherent cultures. For microinjection, cell populations were loaded in borosilicate glass capillary needles (1 mm O.D. × 0.58 mm I.D., Harvard Apparatus, MA, USA) and approximately 250 cells were injected into the perivitelline space of zebrafish embryos, using an IM 300 microinjector (Narishige, London, UK) with an output pressure of 10 psi and 0.03 ms injection time. After injection, embryos were examined for the presence of a fluorescent cell mass at the injection site, and the absence of tumor cells in the circulation was verified. Embryos showing tumor cells in circulation after injection were discarded and not considered for analysis. Xenotransplanted embryos were then transferred to fresh PTU-containing E3 water and placed into a 34 °C incubator for up to three days post injection. Zebrafish embryos were photographed at different time points with a fluorescent microscope DMi8 (Leica) to determine the survival and proliferation of disseminated tumor cells. Data are representative of three or more independent experiments, with more than 30 embryos per group. Experiments were discarded when the survival rate of the experimental groups was less than 70%.

For the CTC and CTC-cluster micromanipulation into the zebrafish, single and cluster suspensions were generated from adherent cultures, and cells were aspirated with the use of a glass capillary inserted into the capillary holder of a manual CellTram Oil (Eppendorf, Hamburg, Germany) micromanipulator. Then, cells were released into the pericardial space of the zebrafish embryo and followed on time by fluorescence microscopy, to determine single CTC and CTC-cluster survival. Four independent experiments were done with at least five fish per experimental group (Single CTC vs. CTC-cluster).

### 4.7. Analysis of Cell Dissemination Pattern in the Zebrafish

The dissemination pattern on tumor cells injected as single cells and clusters was evaluated based on the presence of arrested cells in three locations corresponding to the different vessels of the fish tails. The three different locations considered were: Ventral, involving the caudal vein (CV), posterior cardinal vein (PCV) and dorsal aorta (DA); lateral, involving the segmental artery (SA), and the segmental vein (SV); and Dorsal, involving the dorsal longitudinal anastomotic vessel (DLAV). See schematic representation in Figure S2b. Zebrafish embryos were examined with a fluorescent microscope DMi8 (Leica) to determine tumor cell dissemination.

### 4.8. Fluid Shear Stress (FSS) Assay

Cell suspensions (5 × 10^5^ cells/mL in a total volume of 4 mL) were loaded into a 5 mL syringe (NORM-JECT) attached to a 30-gauge needle. The syringes were loaded into a syringe pump (Harvard Apparatus, Holliston, MA, USA), which generates shear forces in order to mimic the mechanical stress that tumor cells undergo into the bloodstream. It was considered one passage (P) when the entire content of the syringe passed through the 30-gauge needle. Each cell suspension was passed through the needle at a flow rate of 4.45 mL/min (1840 dyn/cm^2^) for a total of 10 passages, with a period of 2 min resting between passages. Aliquots of 100 µL were taken from the cell suspensions before the first passage (P0), as well as after even passages (2nd, 4th, 6th, 8th, and 10th) to assess cell viability, right after finishing the assay (0 h) and after culturing the cells for 24 h. Cell viability was determined by incubating the cell with a luciferin solution (150 μg/mL; Perkin Elmer, Waltham, MA, USA) and quantification of the bioluminescent signal emitted in an EnVision plate reader (Perkin Elmer, MA, USA).

### 4.9. Mouse Lung Colonization Assay and Histological Analyses

Female Scid-Beige mice from the Barcelona Biomedical Research Park (PRBB, Barcelona, Spain) were kept in pathogen-free conditions at the animal facility of the Center for Research in Molecular Medicine and Chronic Diseases (CiMUS, University of Santiago de Compostela), and used at 10 weeks of age. Animal care was handled in accordance with CiMUS guidelines (ES150780275701), and the experimental procedures were approved by the Animal Experimentation Ethical Committee of the University of Santiago de Compostela (15010/2019/002). Tumor growth and metastases formation was monitored by bioluminescence imaging with the IVIS in vivo image system (Perkin Elmer, MA, USA) after intraperitoneal luciferin injection (150 mg Luciferin/kg body weight; Perkin Elmer, MA, USA). MDA-MB-231^eGFP-Luc^ cells (5 × 10^5^ cells) prepared as single cell or cluster suspensions were resuspended in 100 µL of PBS and injected into the lateral tail vein of the mice. One hour after injection, the presence of the cells in the mice lungs was determined by bioluminescence imaging with the IVIS in vivo image system (Perkin Elmer) after intraperitoneal luciferin injection (150 mg Luciferin/kg body weight; Perkin Elmer). Metastases growth was evaluated every 3–4 days for 18 days, time at which mice were sacrificed and lungs collected for bioluminescence image and histological analyses. The lungs were fixed in 10% *v/v* formalin and embedded in paraffin. Sections were made for hematoxylin-eosin and immunohistochemistry for AE1/AE3 keratins (Roche diagnostics, Basel, Switzerland).

Metastasis quantification was reached through review of 17 digitalized slides. We used 3DHISTECH’s SlideViewer version 2.5 (3DHISTECH Ltd. Budapest, Hungary). During this process, each metastatic focus was associated with a marker from the Marker Counter tool, which allowed us to obtain the total marker count and, thus, the total number of metastases per slide.

### 4.10. Isolation of Injected Tumor Cells from Zebrafish Embryos

Zebrafish embryos xenotransplanted with fluorescent single tumor cells (ZF-single) or clustered tumor cells (ZF-clusters) were euthanized with tricaine overdose and the tails were physically separated from the rest of the embryos body. Tails corresponding to each experimental group were pooled together in a 1.5 mL tube, washed twice in PBS (Ca^2+^ and Mg^2+^ free), and enzymatically/mechanically digested by incubating with 100 ul of a solution containing 0.25% *v/v* Trypsin-EDTA in DMEM heated at 37 °C and up and down pipetting for a period of 10–15 min, until tails fragments are no longer visible. Trypsin was neutralized with 400 µL DMEM + 10% FBS (*v/v*) and cells were centrifuged at 700 g for 5 min. Finally, cells were washed in PBS and pelleted for RNA extraction.

### 4.11. Gene Expression Analysis

Gene expression assays were performed by RT-qPCR. RNA was extracted from the pooling of cell suspensions, from four independent experiments, obtained by the digestion of ZF-single and ZF-cluster, respectively, using the RNeasy Micro Kit (Qiagen, Hilden, Germany), following the manufacturer’s instructions. RNA was quantified using the spectrophotometer NanoDrop 2000 (Thermo Fisher Scientific, Waltham, MA, USA) and the reverse transcription process (RT-PCR) was performed using the SuperScript™ III Reverse Transcriptase (Thermo Fisher Scientific), according to the specifications of the manufacturer: 25 °C for 5 min, 50 °C for 2 h and 70 °C for 15 min. One microliter of the cDNA generated was pre-amplified using the Master Mix TaqMan (Applied Biosystems, Waltham, MA, USA) using the following protocol: 95 °C for 10 min and 14 cycles of 95 °C for 15 s and 60 °C for 4 min. The resulting cDNA was diluted 1:10 and further amplified with TaqMan^®®^ probes (Thermo Fisher Scientific, Waltham, MA, USA) and TaqMan^®®^ Universal PCR Master Mix (Life Technologies, Carlsbad, CA, USA) in a LightCycler^®®^ 480 System (Roche Life Science, Barcelona, Spain). The PCR amplification reaction was performed as follows: denaturation at 95 °C for 10 min, followed by 45 cycles of 95 °C for 10 s, 60 °C for 30 s, and 72 °C for 10 s. Gene expression levels are shown normalized by the ΔCt method, using the average expression of glyceraldehyde-3-phosphate dehydrogenase gene (GAPDH) and Beta-2-Microglobulin (B2M) as housekeeping and reference genes. Each sample was profiled in two technical replicates and in duplicates for expression of BAX, CCND1, CDK4, CD44, ITGA6, CTNNB1, E2F4, JUP, MKI67, PLAU, and VIM.

### 4.12. Data Analysis

In vitro and in vivo assays images generated by microscopy were analyzed by the LAS X software (Leica Microsystems, L’Hospitalet de Llobregat, Spain) and the free-software ImageJ (NIH Image, Bethesda, MD, USA). Fluorescence images from zebrafish experiments were analyzed using the free-software Quantifish (Zebrafish Image Analyser). Quantification, statistical analysis as well as graphic representation of each assay was made using the software Prism 6 (GraphPad Software, San Diego, CA, USA).

## 5. Conclusions

The combination of in vitro and in vivo animal models can faithfully represent the biological context of CTCs, providing reliable data about the behaviors of these circulatory cells. In particular, the zebrafish embryo as a model organism offers the possibility to deeply study specific biological features of CTCs and CTC-clusters regarding the metastasis process.

## Data Availability

Not applicable.

## Supplementary Materials

The following are available online at https://www.mdpi.com/article/10.3390/ijms22179279/s1, Table S1. Panel of genes analyzed by RT-qPCR using Taqman probes. Figure S1: Phenotypic characterization of MDA-MB-231 and MCF7 BC cells in different assays. Figure S2: Characterization of MDA-MB-231^eGFP-Luc^ cells injected as single cells or as clusters in the zebrafish embryo., Figure S3: Evaluation of the resistance of single cells and clusters to shear stress in the zebrafish and in vitro. Figure S4: Gene expression analysis of tumor cells isolated from ZF-single and ZF-cluster.

## Abbreviations

BAX	BCL2 Associated X.
B2M	Beta-2-Microglobulin.
CCND1	Cyclin D1.
CD44	CD44 gene.
CTCs	Circulating tumor cells.
CTNNB1	Catenin Beta 1.
EDTA	Ethylenediamine tetraacetic acid.
FBS	Fetal Bovine Serum.
GAPDH	Glyceraldehyde-3-Phosphate Dehydrogenase.
eGFP	Enhanced green fluorescent protein.
JUP	Junction Plakoglobin.
MKI67	Marker of Proliferation Ki-67.
PFA	Paraformaldehyde.
PTU	N-Phenylthiourea.
VIM	Vimentin.
BC	Breast cancer.
CDK4	Cyclin-dependent kinase 4.
CTC	circulating tumor cells.
DMEM	Dulbecco’s Modified Eagle Medium.
E2F4	E2F Transcription Factor 4.
FSS	Fluid shear stress
ITGA6	Integrin Subunit Alpha 6.
Luc	Luciferase.
PBS	Phosphate Buffered Saline.
PLAU	Plasminogen Activator, urokinase.
RT-qPCR	Reverse transcription-polymerase chain reaction.
