# Supplementary material for: Dissecting Breast Cancer Circulating Tumor Cells Competence via Modelling Metastasis in Zebrafish
Source: Int J Mol Sci. 2021 Aug 27;22(17):9279. doi: 10.3390/ijms22179279 (PMC8431683; doi:10.3390/ijms22179279)
Supplement: Supplementary file 1 [file ijms-22-09279-s001.zip › ijms-1318108-supplementary.pdf]

International Journal of
Molecular Sciences

MDPI

Table S1. Panel of genes analyzed by RT-qPCR using Taqman probes.

Gene	Taqman Assay
B2M	Hs00187842_m1
BAX	Hs00180269_m1
CCND1	Hs00765553_M1
CD44	Hs01075861_m1
CD49f (ITGA6)	Hs01041011_m1
CDK4	Hs01565683_g1
CTNNB1	Hs00355049_m1
E2F4	Hs00608098_m1
GAPDH	Hs99999905_m1
JUP	Hs00158408_m1
MKI67	Hs01032443_m1
PLAU	Hs01547054_m1
VIM	Hs00958116_m1

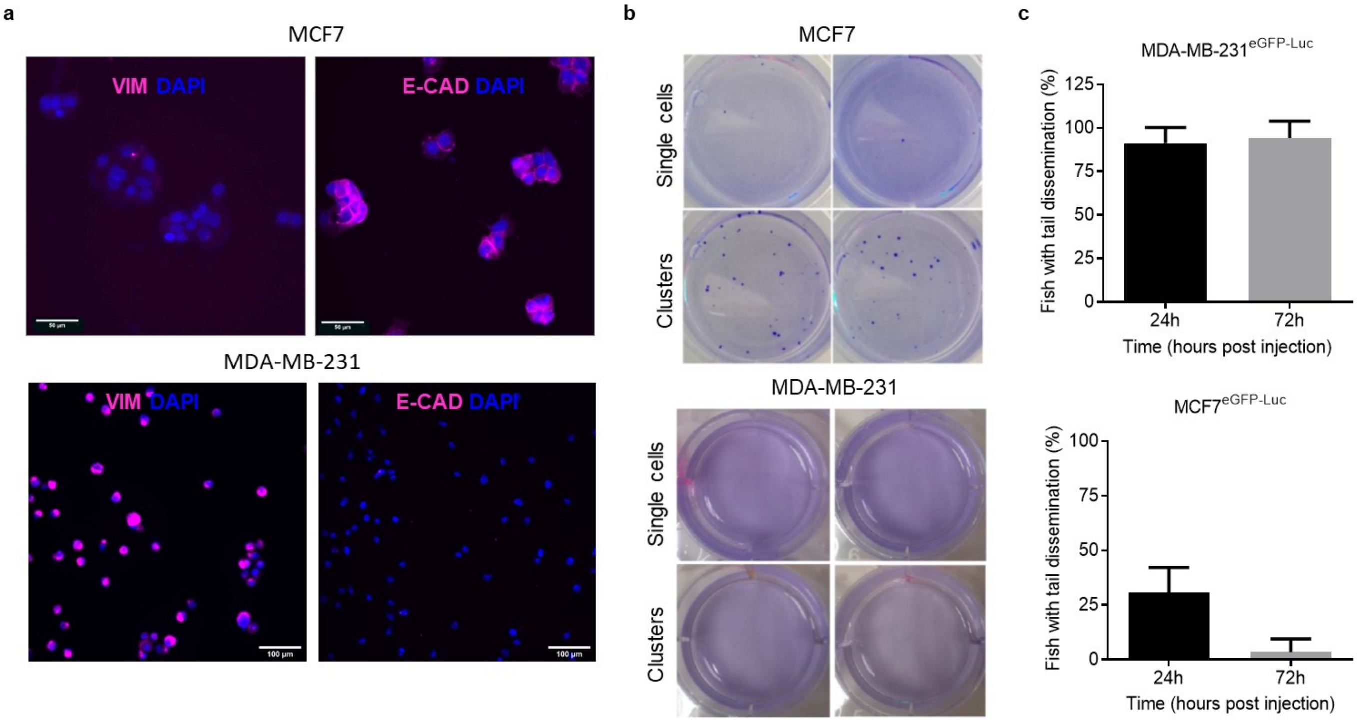

Figure S1. Phenotypic characterization of MDA-MB-231$^{eGFP\text{-}Luc}$ and MCF7$^{eGFP\text{-}Luc}$ BC cells in different assays. **(a)** Expression of Vimentin and E-cadherin in MCF7 and MDA-MB-231 cell lines assessed by immunofluorescence. Scale bars 100 µm. **(b)** Representative images of colonies formed by single and cluster cell populations of MCF7 and MDA-MB-231 cell lines. **(c)** Percentage of zebrafish injected with clusters of MDA-MB-231$^{eGFP\text{-}Luc}$ and MCF7$^{eGFP\text{-}Luc}$ cells showing dissemination of tumor cells to the tail.

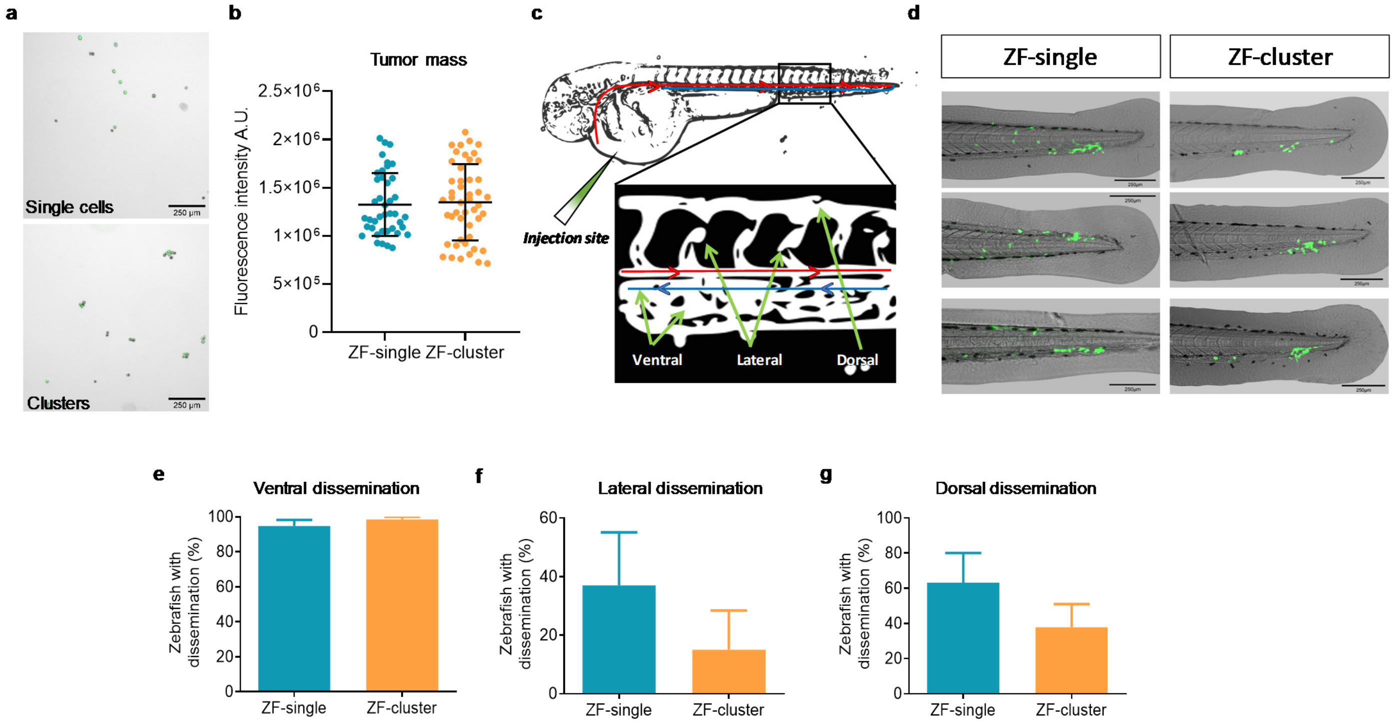

Figure S2. Characterization of MDA-MB-231$^{eGFP-Luc}$ cells injected as single cells or as clusters in the zebrafish embryo. **(a)** Representative images of single and cluster cell suspensions injected into the zebrafish embryo. **(b)** Quantification based on the fluorescence signal of the tumors generated in ZF-single and ZF-clusters at the site of injection. **(c)** Schematic representation of the zebrafish embryo circulation and the different locations evaluated for cell arrest: dorsal, lateral and ventral. **(d)** Representative images of the dissemination pattern of single and cluster cells in the zebrafish embryo tails. Percentage of embryos injected with single and cluster cell suspensions showing cell dissemination at ventral **(e)**, lateral **(f)**, and dorsal **(g)** areas of the fish tails. Scale bars 250 μm.

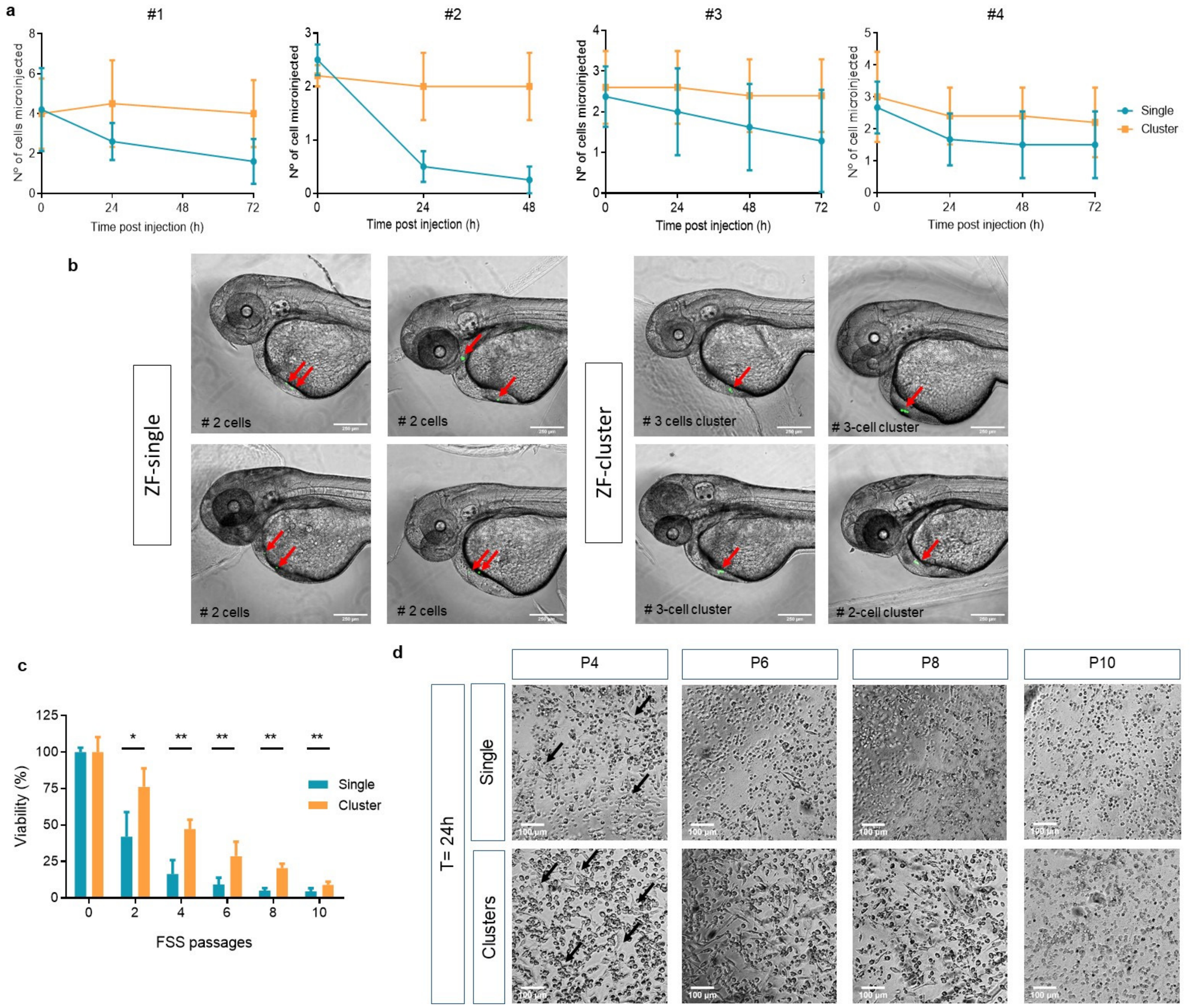

Figure S3. Evaluation of the resistance of single cells and clusters to shear stress in the zebrafish and in vitro. **(a)** Individual experiments showing the specific number of cells microinjected into the pericardial cavity of the embryos either as single cells or as clusters. **(b)** Representative images of small numbers of single and cluster cells microinjected in the embryo. Red arrows show tumor cells. Scale bars 250 µm. **(c)** Cell viability of single and cluster cell populations after being subjected to different passages of fluid shear stress (FSS). **(d)** Representative images of single and cluster populations after four, six, eight and ten FSS passages. Arrows show tumor cells adhered to the well surface. Scale bars 100 µm. * $p < 0.05$, ** $p < 0.01$.

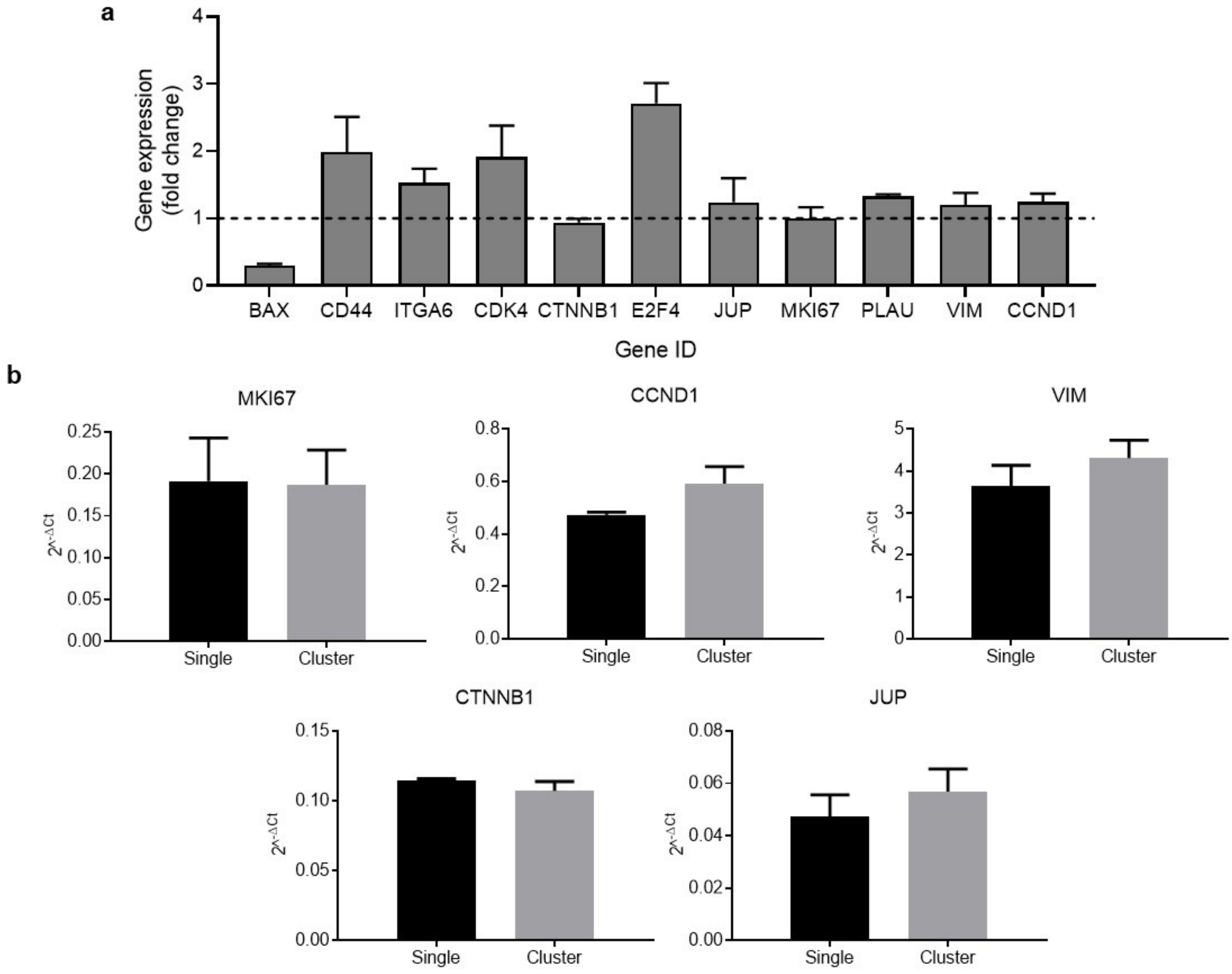

Figure S4. Gene expression analysis of tumor cells isolated from ZF-single and ZF-cluster. **(a)** Relative gene expression levels of cells isolated from ZF-cluster to ZF-single. **(b)** Gene expression levels expressed as $2^{-\Delta CT}$ of MKI67, CCND1, VIM, CTNNB1, and JUP in both cell populations relative to the average expression of GAPDH and B2M.
